# Role of tumor suppressor p53 and micro-RNA interplay in multiple myeloma pathogenesis

**DOI:** 10.1186/s13045-017-0538-4

**Published:** 2017-10-26

**Authors:** Jahangir Abdi, Nasrin Rastgoo, Lihong Li, Wenming Chen, Hong Chang

**Affiliations:** 1 Division of Molecular and Cellular Biology, Toronto General Research Institute, Toronto, Canada; 2 Department of Laboratory Medicine & Pathobiology, University of Toronto, Toronto, Canada; 3 Department of Hematology, Beijing Chaoyang Hospital, Capital Medical University, Beijing, China; 4 Department of Laboratory Hematology and Medical Oncology, University Health Network, 200 Elizabeth Street, 11E-413, Toronto, ON M5G 2C4 Canada

**Keywords:** p53, Micro-RNA, Myeloma

## Abstract

The molecular mechanisms underlying dysregulated wild type (wt) p53 in multiple myeloma (MM) have been subjects of intense investigation for years. Indeed, correlation of rarely occurring *TP53* gene mutations or deletions with adverse clinical outcomes in MM patients is strongly established, while in majority of cases wtp53 seems to be non-functional or dysregulated bearing a high clinical impact. Interestingly, findings from recent investigations show that micro-RNAs (miRNAs) may contribute to suppression of wtp53 in MM, as they are now known to function as key regulatory elements in the p53 network. This area is shedding new light on understanding the biologic effects of dysregulated p53 in MM pathogenesis especially drug resistance. miRNAs such as miR-125b (oncomiR) or miR-34a (tumor suppressor-miR) can be negative or positive regulators of wtp53 function, respectively, with specific effects on MM cell viability. On the other hand, our knowledge of miRNA interaction with mutant (mt) p53 in MM, which is rather related to disease progression and resistance to therapy, is limited which demands in-depth exploration. Here, we will put forward the current knowledge on miRNA-p53 interaction in MM and its role in MM pathogenesis including drug resistance. We will also highlight the pre-clinical approaches for therapeutic application of miRNAs targeting p53 pathway.

## Background

In multiple myeloma (MM), p53 is frequently dysregulated culminating in abnormal function of p53 signaling pathway and its related downstream targets [1], while mutations or deletions in *TP53* gene are rare phenomena in MM, with approximate rates of 3 or 10%, respectively, at diagnosis as reported by us and others [2–5]. These observations reflect the fact that inactivation of a wild-type functional p53 (wtp53) could be the major contributor to dysregulation of p53 pathway, hence MM pathogenesis especially drug resistance. Accordingly, extensive research has been focusing on deciphering the mechanisms of dysregulation of p53 pathway and related targets. As reported by us [6–8] and other groups [9, 10], non-genotoxic targeting of the pathway using small molecules indicates that negative regulators of p53 including MDM2 contribute largely to p53 pathway dysregulation in MM. However, elucidation of the mechanisms underlying p53 pathway dysregulation in MM demands more detailed studies.

Micro-RNAs (miRNAs) are small non-coding RNAs (~ 20–24 nt in length) that are involved in the post-transcriptional control of gene expression. miRNAs generally bind to target mRNAs through their 3′ untranslated regions (UTRs) and recruit the RNA-induced silencing complex (RISC), which mediates the inhibition of translation and the degradation of the respective mRNA [11]. It is estimated that > 60% of human protein coding genes may be subject to regulation by miRNAs [12]. Among these targets, the tumor suppressor gene *TP53* is of particular importance as it can regulate expression and processing of miRNAs and the mRNA of *TP53* can be a direct target of miRNAs [13]. Interestingly, it has been suggested that miRNAs may have evolved to provide the cells with the ability to effectively deal with stress [14, 15]. This concept further supports the notion that p53 stress response pathway would have a strong interrelationship with miRNAs. Moreover, a great body of evidence has now established miRNAs as critical players in MM pathogenesis, progression, and therapy resistance [16–19].

In recent years, some studies in MM have taken advantage of the role of miRNAs in attempts to better understand the mechanisms underlying p53 dysregulation and its contribution to drug resistance, the critical aspect of MM pathogenesis. However, these studies focused mainly on interaction of wtp53 with miRNAs in MM; thus, whether/how miRNAs interact with mutant p53 (mtp53) to possibly explain the resistance to therapy or relapse in MM patients carrying mt53 is still not clear. This review will discuss the current understanding of p53-miRNA interaction and its role in MM pathogenesis especially drug resistance. Potential therapeutic applications of miRNAs which targetp53 pathway will also be highlighted.

### miRNAs in a p53 network: control of MM cell viability and drug response

The tumor suppressor p53 is inactivated in almost all human cancers while only 50% harbor *TP53* mutations, particularly in MM where this rate may be as low as 3% early at diagnosis. Moreover, mono allelic deletion of *TP53* is detected only in approximately 10% of MM patients at diagnosis [2–5]. These observations highlight the fact that suppressed p53 or frequent dysregulation of p53 signaling pathway in MM emerges even in the face of a functional wtp53. Interestingly, as demonstrated by several studies in recent years in MM [20, 21] and other cancers [22], miRNAs may have an essential role in lowering p53 expression or activity. Hundreds of genes and their products establish a complex network comprising p53, its regulators, and regulated genes which collectively help p53 maintain its proper function [1]. Recent studies have demonstrated that miRNAs interact with p53 and its network at multiple levels. Thus, p53 can regulate the transcription expression and the maturation of a group of miRNAs, while miRNAs can regulate the activity and function of p53 through direct repression of p53 or its regulators. These findings indicate that miRNAs are important components in the p53 network and add another level of complexity to the p53 network [23].

Understanding the mechanisms behind dysregulation of functional p53/p53 signaling pathway in MM will shed new light on pathogenesis particularly drug resistance and refractoriness of the disease. Due to their versatility in gene regulation, miRNAs have recently been identified as factors capable of modulating viability and drug response of MM cells in relation to their p53 status. The mutual functional link between miRNAs and p53 in cancers including MM has provided some important clues to the mechanisms underlying p53/p53 pathway dysregulation which will in turn open new venues to MM therapy. In two separate sections as follows, we will discuss the biologic outcomes of such interaction for two categories of miRNAs, negative and positive regulators of p53 function.

#### iRNAs as positive regulators of wtp53 in MM cells: a suppressed positive feedback loop threatens viability

**Fig. 1:**
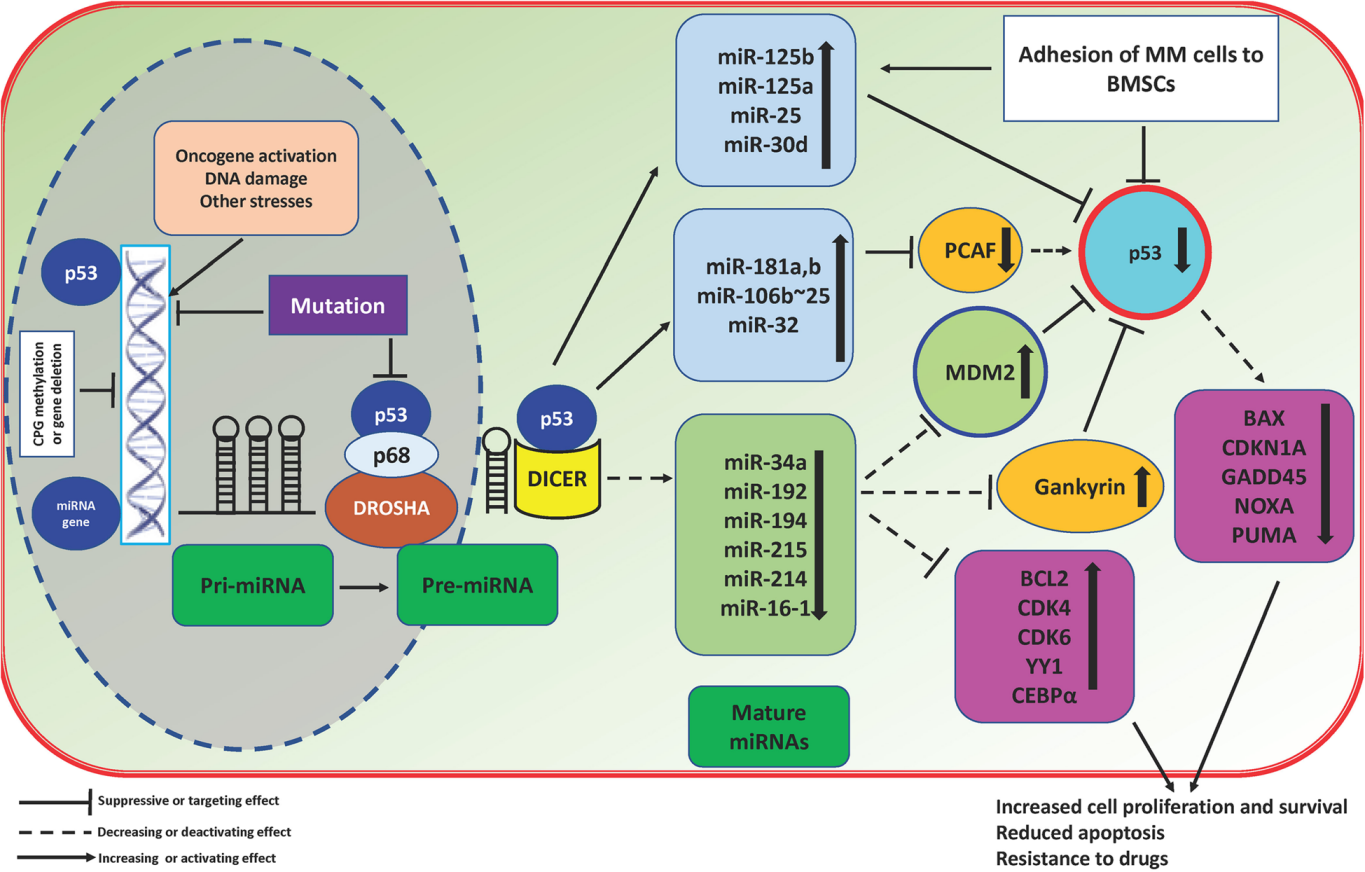
Schematic mechanism of p53-miRNA interaction in MM. TS-miRs known to be regulated by wtp53 (e.g., miR-192) target negative regulators of wtp53 like MDM2 and gankyrin or target cell cycle-related/anti-apoptotic proteins to enhance tumor suppressor activity of wtp53. However, this scenario is compromised in MM context possibly due to epigenetic silencing of TS-miRs or wtp53 and also due to upregulation of negative regulators. Therefore, the outcome will be a suppressed wtp53 which ends in downregulation of p53 downstream targets such as PUMA, BAX, and NOXA, maintenance of MM cells survival, and probably resistance to anti-myeloma drugs. On the other hand, oncomiRs (e.g., miR-125b) negatively regulate p53 either by directly targeting p53 or by targeting positive regulators of p53 (such as PCAF) which culminates in the same outcomes as above. Of note, wtp53 can also regulate miRNA biogenesis by association with DROSHA or DICER, a function which is completely antagonized by mtp53. In MM, mtp53 may function as an oncogene by interfering with biogenesis of TS-miRs which could partly explain the resistance of mtp53 phenotypes to anti-myeloma drugs (however, this concept demands in-depth investigation in MM)

It is well established that p53 as a transcription factor can directly regulate the expression of a growing number of miRNAs (Table 1). This indicates that p53 can transactivate tumor suppressor (TS)-miRs or repress some of the oncomiRs. miRNAs transactivated by p53 mainly target the anti-apoptotic and anti-proliferative genes, hence boosting the tumor suppressor activity of p53, or they can even regulate p53 itself in a positive feedback loop. On the contrary, miRNAs repressed by p53 may target the tumor suppressor or pro-apoptotic genes leading to dampening tumor suppressor activity of p53 [24] (concept illustrated in Fig. 1).

**Table 1:** miRNAs which function as negative or positive regulators of wtp53

miRNA	Expression pattern	P53 status of the HMCLs used	Functional responses and targets	Refs.
miR-125b	Upregulation (UR)	Wild type (wt)	MM cells overexpressed miR-125b when exposed to DEX leading to reduced apoptosis by targeting p53. miR-34a which targeted SIRT1 was also induced by DEX, thus maintained deacetylation and inactivation of p53. Inhibition of miR-125b enhanced p53 expression, suppressed SIRT1, and increased DEX-induced apoptosis	[20]
miR-125a	UR	wt	miR-125a-5p mimics downregulated the expression of TP53, BAX, MDM2, CDKN1A, and GADD45. p53 was directly targeted by miR-125a-5p. Moreover, inhibition of miR-125a-5p dampened cell growth, increased apoptosis, and reduced cell migration	[21]
miR-34a	Downregulation (DR)	wt and mutant (MT)	Synthetic miR-34a downregulated canonic targets BCL2, CDK6, and NOTCH1 at both the mRNA and protein level. In a xenograft model of mtp53, miR-34a mimics also reduced tumor growth	[27]
miR-34a	DR	wt and MT	Overexpression of miR-34a reduced the level of Bcl-2, CDK4, CDK6, CEBPα, and YY, sensitized them to BTZ, and reduced tumor growth in vivo	[29]
miR-214	DR	wt	miR-214 overexpression in H929 cells resulted in suppression of PSMD10 and ASF1B. Inhibition of gankyrin increased P53 mRNA levels and subsequently upregulated CDKN1A (p21Waf1/Cip1) and BAX transcripts, which are direct transcriptional targets of p53	[32]
miR-192/194/212	DR	wt and MT	MDM2 inhibitor nutlin-3a upregulated p53 and 3 p53-inducible miRNAs, miR-192/194/215. Ectopic expression of these miRNAs in wtp53 HMCLs upregulated CDKN1A but not in mtp53 lines. Furthermore, while wtp53 HMCLs were sensitive to miRNA upregulation, mtp53 cells showed some level of resistance	[38]
miR-25 and miR-30d	UR	wt	Inhibition of miR-25 or miR-30d increased the endogenous protein levels of p53, Bax, p21, and PUMA and induced apoptosis, while ectopic overexpression of these miRNAs decreased p53, p21, and GADD45 and reduced apoptosis and cell cycle arrest. p53, p21, and Bax were decreased even in the presence of the genotoxic agent etoposide	[39]
miR-106b~25 cluster, miR-32, miR-181a/b	UR	wt	These miRNAs targeted p300-CBP-associated factor (PCAF), an activator of wtp53. Inhibition of all these miRNAs highly upregulated TP53 following exposure to UV light	[54]

miRNAs which activate p53 act indirectly by interacting with p53-regulating factors. These miRNAs include miR-122, 34, 29, 192, 194, 215, and 605 which activate (regulate abundance/activity of) p53 by binding to the 3′UTR of factors like MDM2, SIRT1, and YY1 [13]. The first indication of a miRNA transcriptionally regulated by wtp53 was reported when several studies found miR-34 family members miR-34a/b/c to be directly regulated by wtp53 [25, 26]. miR-34 family members are well-known TS-miRs which repress the expression of several targets involved in regulation of cell cycle and proliferation, like BCL-2, Notch1, cyclin E2, cyclin-dependent kinases 4 and 6 (CDK4 and CDK6), in MM [27] and other cancers [26]. Moreover, miR-34a has been identified to be hypermethylated at its promoter region in MM [28]. Notably, in one study, the expression of miR-34a was correlated with *TP53* mutational status and del17p.13 (by fluorescent in situ hybridization (FISH)) in human myeloma cell lines (HMCLs) [29], consistent with the notion that miR-34a is transactivated by wtp53 [30]. Mutations in exons 5 and 8 of *TP53* were identified in all HMCLs with low miR-34a. In particular, del17p.13 by FISH was noted in KMS11 cells (*TP53* ^*NULL*^), consistent with their very low or undetectable miR-34a. Furthermore, overexpression of miR-34a in KMS11 and OPM2 cell lines reduced the level of Bcl-2, CDK4, CDK6, CEBPα, and YY1, sensitized them to bortezomib (BTZ) and reduced tumor growth in vivo. Anti-myeloma activity of miR-34a was reported in another study showing the killing effects of miR-34a mimics in xenograft harboring *TP53*-mutated MM cells [27]. Additionally, low miR-34a level was reported to be correlated with low p53 expression and resistance to fludarabine in CLL [31], further supporting the role of miR-34a in regulation of p53-mediated tumor cell death.

miR-214 is another miRNA which has been reported to positively regulate p53 abundance and function in wtp53 HMCLs [32]. Ectopic expression of miR-214 induced apoptosis and reduced cell growth in wtp53-carrying H929 cells and suppressed PSMD10 (which encodes the oncoprotein gankyrin, a p53 negative regulator) and ASF1B (a histone chaperone required for DNA replication), which were found to be direct targets of miR-214. Inhibition of gankyrin increased *P53* mRNA levels and subsequently upregulated CDKN1A (p21Waf1/Cip1) and BAX transcripts, which are direct transcriptional targets of p53. These results indicate that miR-214 functions as a tumor suppressor in MM by positive regulation of p53 and inhibition of DNA replication. Of note, high level of miR-214 in the serum of MM patients was reported to be correlated with aggravated bone disease and poor outcome [33]. However, this study did not provide any evidence that miR-214 displays the same expression and function (as an oncomiR) in the tumor cells from the bone marrow or in the HMCLs. Further, they could not find any correlation of high serum miR-214 with MM patients’ response to BTZ and argued that high miR-214 in the serum was much more associated with myeloma bone destruction rather than tumor burden. Additionally, miR-214 regulated cancer stemness in solid tumors by targeting p53 and Nanog [34], suggesting context-dependent expression and function of miR-214.

A cluster comprising miR-192, miR-194, and miR-215 has been identified as p53-induced miRNAs in several cancer types including MM [35–38]. Pichiorri et al. [38] provided evidence that miR-192, miR-194, and miR-215 are transcriptionally induced by p53 and they, in turn, activate p53 indirectly by targeting MDM2 leading to p21 upregulation and induced apoptosis and cell cycle arrest in wtp53 HMCLs. Introduction of these miRNAs into wtp53 HMCLs (MM.1S, H929, KMS28BM) led to upregulation of *CDKN1A* and downregulation of MDM2 in mtp53 cell lines but no change at *TP53* mRNA. Interestingly, miRNA transfection in wtp53 cells with higher MDM2 level (MM.1S and H929) induced G0/G1 arrest, while in wtp53 cells with lower MDM2 level (KMS28BM), it triggered sub-G0 accumulation (apoptotic cell death). These findings clearly indicate that the p53-induced miR-192, miR-194, and miR-215 control MM cell viability by targeting MDM2.

#### miRNAs as negative regulators of wtp53 function in MM cells: a negative feedback loop threatens viability

Several miRNAs have been described to negatively regulate p53 by directly binding to its 3′UTR: miR-25 and miR-30d [39], miR-125b, [40] miR-504, [41] miR-380-5p, [42] miR-92, and miR-141 [43]. However, only a few of them have been functionally explored in relation to p53 in MM (Table 1). Kumar et al. [39] observed that miR-25 and miR-30d were overexpressed around 6.3- and 11.2-fold, respectively, in plasma cells from MM patients compared with those from healthy donors. Inhibition of miR-25 or miR-30d increased the endogenous protein levels of p53, Bax, p21, and PUMA and induced apoptosis, while ectopic overexpression of these miRNAs decreased p53, p21, and GADD45 and reduced apoptosis and cell cycle arrest. The authors further observed that p53, p21, and Bax were decreased even in the presence of the genotoxic agent etoposide implying a suppressive role for miR-25/miR-30d in p53-mediated apoptosis.

mir-125b is the most important p53-targeting oncomiR to be studied in MM pathogenesis especially drug resistance because (a) it has been shown to play an eminent role in normal lymphopoiesis or myelopoiesis and also to function as an oncomiR in hematologic malignancies at least by targeting p53 [44], (b) latter function of miR-125b is further supported by the observations that it also targets other components of the p53 pro-apoptotic network including BAK1, PUMA, BMF, TRP53INP1, and Kruppel-like factor 13 (KLF13) [45, 46], (c) miR-125b proved to be a leukemogenic oncomiR in mouse models by causing acute myelogenous leukemia (AML) and acute lymphoblastic leukemia (ALL) phenotypes [47, 48], and (d) miR-125b was reported to be associated with drug resistance in ALL [49, 50]. Megan et al. described a mechanism whereby MM cells overexpressed the oncogenic miR-125b when exposed to dexamethasone (DEX) leading to reduced apoptosis, and they further showed direct targeting of *TP53* mRNA by miR-125b [20]. Interestingly, miR-34a which targeted SIRT1 deacetylase was also induced by DEX, hence allowing maintenance of deacetylation and inactivation of p53. Strikingly, inhibition of miR-125b enhanced expression of p53, repressed expression of anti-apoptotic SIRT1, and, importantly, significantly enhanced DEX-induced cell death in MM cells. These findings suggest that resistance against chemotherapy in MM cells works greatly via the p53/miR-34a/SIRT1 signaling network which provides a platform for therapeutic intervention.

miR-125a, which shares the same seed sequence with miR-125b [51], has also been reported to show the same expression pattern and function as miR-125b in hematologic malignancies including diffuse large B cell lymphoma (DLBCL) [52]. In MM, Leotta et al. [21] showed that when MM cells were transfected with miR-125a-5p mimics, expression of *TP53* gene and p53 pathway-related genes such as BAX, MDM2, CDKN1A, and GADD45 was downregulated, and p53 was found to be a direct target of miR-125a-5p. Moreover, inhibition of miR-125a-5p expression in wtp53 MM cells dampened cell growth, increased apoptosis, and reduced cell migration. These findings strongly support the oncogenic function of miR-125a-5p in MM cells. Notably, miR-125a was associated with resistance to daunorubicin in leukemia cell lines further suggesting its role in drug resistance as an oncomiR [53].

It has also been shown that in MM cells, wtp53 can be indirectly targeted by miRNAs. Pichiorri et al. found that miR-106b~25 cluster, miR-32, and miR-181a/b were overexpressed in MM cells relative to normal controls [54]. These miRNAs targeted p300-CBP-associated factor (PCAF), a histone acetyltransferase which reversibly acetylates p53, hence an activator of wtp53. They also observed that PCAF expression was almost absent (10-fold less than in control) in 10 out of 15 HMCLs, whereas the remaining 5 cell lines displayed very low expression. In an interesting approach, they transfected MM.1S cells (wtp53) with anti-miR-181s, anti-miR-106b, anti-miR-25, and anti-miR-32, exposed the cells to UV radiation, and measured the expression of p53 and PCAF by q-RT-PCR. After UV treatment, PCAF was accumulated and p53 mRNA expression was increased sixfold after nucleoporation with all antagomiRs simultaneously. Taken together, these findings show that the miR-106b-25 cluster, miR-32, and miR-181a and b target PCAF and through this gene, indirectly control p53 activity and apoptosis in MM cells.

It is important to note that the p53-miRNA axis may also be modulated by exogenous agents which can regulate p53-targeting miRNAs. For instance, Gordon et al. [55] reported that some environmental carcinogens may contribute to MM oncogenesis by upregulating p53-targeting miRNAs (oncomiRs); however, this notion requires further support.

#### Interaction of miRNAs with mtp53: how does miRNA-p53 axis work in the advanced stages of MM?

Most of the mutations within the *TP53* gene are missense giving rise to a full-length mtp53. This is a unique feature because most other tumor suppressor genes are frequently inactivated by frame shift or nonsense mutations leading to either production of truncated proteins or complete elimination of the corresponding gene products [56]. In MM, dysregulation of p53/p53 pathway cannot be due to just *TP53* mutations as they occur quite rarely at diagnosis, while wtp53 is frequently dysregulated leading to an imbalance in p53 activity which could be generated through post-transcriptional or post-translational mechanisms. Additionally, our major understanding of the miRNA-p53 interaction in MM pathogenesis is derived from the wtp53, i.e., from studies on MM patients at diagnosis or HMCL harboring wtp53. Thus, it seems conceivable that non-mutational dysfunction of wtp53 bears greater association with MM pathogenesis. However, the fact that *TP53* mutations will become more frequent as MM disease progresses cannot be ignored, with their prevalence reaching 25–30% in plasma cell leukemia (PCL) [57, 58] and 80% in HMCLs [59]. Therefore, it is crucial to understand whether mtp53-miRNAs interaction also plays any role in MM pathogenesis especially in the advanced stages of the disease. This concept will also help us to find out if such aberrant axis underlies drug resistance in MM patients who are refractory to treatment or at relapse.

In all studies in MM specifically addressing p53-miRNA interaction, functional role of mtp53 is rather elusive or lacking. *TP53* mutations have been detected in all HMCLs with low miR-34a and especially del17p.13 in KMS11 cells with undetectable miR-34a; however, miR-34a mimics sensitized them to BTZ raising the probability that miR-34 may function in a p53-independent as well, as indicated elsewhere [29]. On the other hand, reintroducing miR-192, miR-194, and miR-215 into wtp53 HMCLs (MM.1S, H929, KMS28BM) led to significant upregulation of *CDKN1A* and suppression of colony formation but not in mtp53 ones (RPMI-8226 and U266) [60]. These findings provide evidence that miR-192, miR-194, and miR-215 function in a manner dependent upon p53 status, hence, most probably in the presence of a functional wtp53. However, it is still not clear whether resistance of mtp53 HMCLs to these miRNAs is due to compromised miRNA-3′UTR interaction, due to mutant 3′UTR sequence of mtp53, or somehow mtp53 interferes with endogenous or introduced miRNA processing. Also, no clues as to whether mtp53 interaction contributes to drug resistance in MM cells.

To address above issues, specific experimental approaches need to be taken: (1) Generating various *TP53* ^*MUT*^ constructs, introducing them into HMCLs with very low or undetectable p53 to make stable expression, followed by analysis of miRNA profiling with or without anti-myeloma drugs. In support of this idea, transfection of HCT116 colon cancer cell line with tumor-derived p53 mutants decreased mature and precursor miRNA levels of miR-16-1, miR-143, and miR-205 [61]. Furthermore, introducing mutant p53R175H into non-small-cell lung cancer cells upregulated miR-128 which in turn sensitized the cells to chemotherapy by suppressing p21 (CDKN1A) [62]. (2) Isolation of primary MM tumor cells from patients at relapse or refractory to treatments for analysis of miRNA expression profiles in the context of their *TP53* status and comparison with samples at diagnosis. (3) Analysis of *TP53* status and its functional features along with miRNA profiling and 3′UTR binding assays in HMCLs with established drug resistance phenotype such as RPMI-8226R5, MM.1R and comparison with their parental lines. The above approaches will help us to understand if mtp53 in MM cells interacts with miRNAs and whether this interaction modulates drug response of the cells.

Of important note, besides regulating the expression of miRNA genes, wtp53 can affect the maturation (processing) of miRNAs as well. It has been shown that wtp53 directly or indirectly (through p68) interacts with DROSHA complex to promote the processing of specific primary transcript miRNAs (pri-miRNAs) to precursor miRNAs (pre-miRNAs) to increase the levels of the mature miRNAs, e.g., miR-16-1 (which is reported to be a TS-miR in MM [63]), but mtp53 suppressed this interaction [61]. Furthermore, p53 can also interact with DICER1 as evidenced by induced expression of p53 and premature senescence in DICER1 deficiency (incomplete miRNA maturation) [64], whereas mtp53 suppresses DICER1 by inhibiting p63 (a member of p53 family) [65]. Indeed, in mice p63 has been shown to regulate the transcription of Dicer [65]; thus, it would be interesting to investigate whether a global decrease in miRNAs occurs in mtp53 expressing MM cells. Surprisingly, low expression of DICER has been associated with shorter survival and poor prognosis in MM [66] and CLL [67]. Moreover, inactivation of p53 was insufficient to allow untransformed B cells and B cell lymphomas to survive without Dicer [68], providing further support that at least in B cell malignancies interaction of p53 with DICER plays critical role in cell survival and drug response.

Taken all together, mtp53 in contrast to the wtp53 adopts an oncogenic function by interfering with miRNA expression and maturation, another mechanism which might underlie resistance to p53-mediated apoptosis in a mtp53-miRNA network in tumor cells. However, whether this phenomenon explicitly happens in MM cells, to explain the resistance to therapy in MM patients carrying mtp53 demands specific research.

#### p53-miRNA-epigenetics: a trio governing MM cell viability

It has been reported that almost all cases of 17p13 deletion in MM are mono-allelic and the remaining allele is rarely mutated [69]. Whether the remaining allele is inactivated by other mechanisms like DNA methylation or MDM2 upregulation leading to suppressed p53 pathway in MM was explored by Teoh et al. [69]. They demonstrated that p53 haplo-insufficiency accounted for the compromised response of MM cells to genotoxic stress when one allele of *TP53* is lost. They treated a panel of HMCLs with different p53 status with the genotoxic agent etoposide and observed that wt/wt cells (H929) are more sensitive to drug-induced apoptosis than wt/- cells (XG6), mtp53 cells (RPMI-8226 and U266) showed negligible cell death, and cells with very low or completely absent p53 (JJN3 and KMS18) were resistant to drug-induced apoptosis. The authors also identified that hypermethylation of p53 promoter is the cause of inactivation of the remaining *TP53* allele in wt/- MM cells. Promoter hypermethylation has also been reported for some p53-induced miRNAs in MM cells, such as miR-34a, miR-192,194,215, which in fact have tumor suppressor activity [28, 38]. While it is conceivable that p53 suppression in MM cells may be partly due to overexpression of some oncomiRs, it remains to be elucidated whether oncomiRs contribute to p53 suppression by triggering promoter hypermethylation in MM. Epigenetic mechanisms may work indirectly on miRNAs as well to affect p53 activity/abundance. It is reported that some oncomiRs like miR-106b~25 cluster and miR-125b which negatively regulate *TP53* gene (see above) are reduced by EZH2 inhibition. This was indicated by the observation that EZH2 indirectly upregulated those miRNAs by suppressing their transactivators [70]. Pichiorri et al. also describe that miR-192, 194, and 215 are epigenetically silenced in MM cells by promoter hypermethylation. Downregulation of these miRNAs leads to increased MDM2 mRNA and protein expression and decreased p53 level. Interestingly, epigenetic silencing of miR-137 in MM cells lead to MDM2-mediated degradation of p53 through upregulation of AURKA which was found to be a direct target of miR-137. Introducing miR-137 mimics into drug-resistant MM cells could re-sensitize them to anti-myeloma drugs by inhibition of p53 degradation and inducing apoptosis [71]. Therefore, it seems conceivable that a combination of epigenetic silencing of p53-activated miRNAs and p53 in the context of myeloma tumor further contributes to dysregulation of p53 pathway and enforced survival of MM cells.

### The effect of bone marrow stroma on p53-miRNA interaction: implication for mechanism of cell adhesion-mediated drug resistance (CAMDR)

**Fig. 2:**
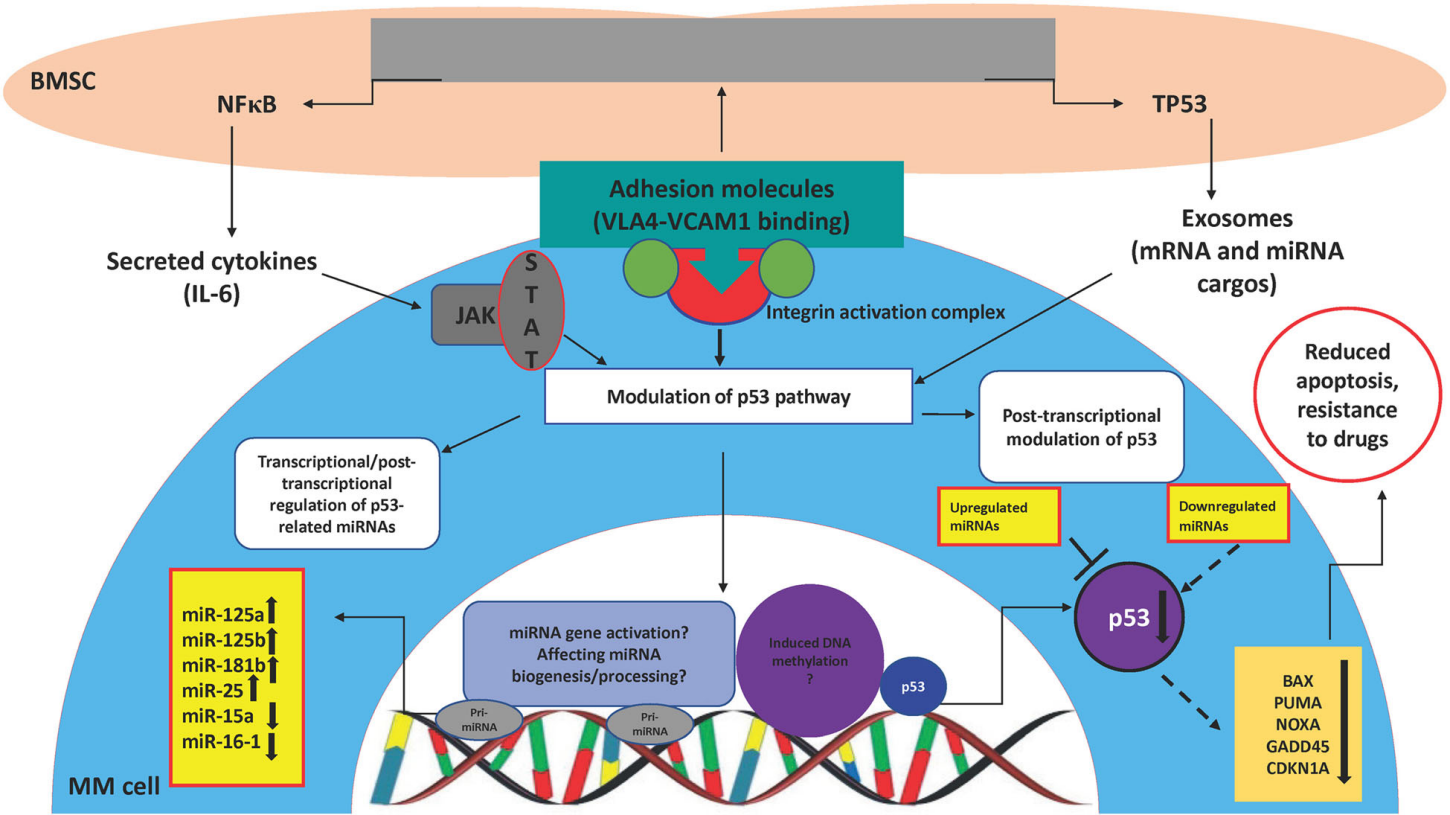
Proposed scheme for the role of BMSCs in modulation of p53-miRNA interaction in association with stroma-induced drug resistance in MM. Following interaction between BMSCs and MM cells, p53 mRNA and protein and some p53-related TS-miRs (miR-15a/16-1) will be downregulated. On the other hand, the expression of some oncomiRs which negatively regulate p53 such as miR-125a, b will be promoted. Interestingly, exosomes released from BMSCs also contain miRNAs which affect p53 pathway. The outcome can be attenuation of p53-mediated apoptotic effects and drug resistance (CAMDR). It is established that integrin activation and cytokine signaling pathways will cross-talk with p53 pathway which ends in modulation of the pathway and also post-transcriptional changes in p53. However, it requires thorough investigations whether pathways activated following BMSC-MM cell interaction somehow impinge on transcriptional modulation of *TP53* or miRNA genes in the nucleus through epigenetic mechanisms, miRNA biogenesis, or gene activation

It is reported that tumor microenvironment may also contribute to non-mutational dysregulation of wtp53 in tumor cells. A few studies have shown that bone marrow stromal cells (BMSCs) can modulate p53-related miRNAs and negatively affect p53 function and expression in MM cells through direct or indirect cell-cell interaction [72, 73]. This phenomenon may be partly supported by the fact that IL-6, the main survival factor for MM cells secreted by BMSCs, indeed suppresses p53 function [74], and integrin activation following tumor cell-stroma adhesion modulates p53 function [75]. Moreover, it is established that integrin engagement following cell-cell adhesion drives cell-cycle machinery [76]. Hence, modulation of p53-targeting miRNAs by BMSCs in MM seems plausible, as they are established as integral part of p53 regulatory network (the stroma concept is illustrated in Fig. 2).

Notably, the few studies performed to date in MM addressing specifically the effect of BMSCs on expression and function of p53 used only wtp53 MM cells. The most probable reason is to understand whether stroma regulates/modulates p53 “with a normal function” in the context of drugs, as it is reported that at advanced stages of the disease (where the rate of p53 mutation will also increase) MM cells may become stroma-independent/unresponsive [77]. Leotta et al. [21] showed that adhesion of wtp53 MM cells to BMSCs strongly upregulated miR-125a-5p level, while reduced p53 expression; however, whether interaction between these two contributes to drug resistance in stroma context was not pursued. Furthermore, in a preliminary work, we showed that when wtp53 harboring MM.1S cells adhered to BMSCs, the mRNA of *TP53*, *BAX*, *NOXA*, MDM2, and *PTEN* was downregulated, while p53-targeting miRNAs, miR-125a, miR-125b, miR-25, and miR-181b were upregulated. On the other hand, some p53-regulated miRNAs such as miR-15a and miR-16 were downregulated. Above modulations happened in the context of BTZ which gives an implication of involvement of p53/miRNA interaction in stroma-induced drug resistance in MM.

Interestingly, recent studies have identified an important role for exosomes secreted from BMSCs in modulation of wtp53 function and in contribution to stroma-mediated drug resistance in MM [78]. These extra cellular vesicles indeed contain mRNAs and miRNAs which through unclarified mechanisms affect various signaling pathways including p53 in MM cells. Strikingly, p53 has been reported to play a key role in exosome secretion [79]. Furthermore, p53 status of stromal cells in tumor environment is reported to influence the drug response of tumor cells [80]; thus, it would be interesting to explore if p53 status in BMSCs from MM patients affects the level of protection for MM cells or their response to anti-myeloma drugs.

Taken together, BMSCs have suppressive effects on wtp53 expression and function probably by modulating p53-related miRNAs. It remains to be elucidated how BMSCs generate such effects; however, induction of an epigenetic silencing mechanism in MM cells may be speculated. A study conducted by Amodio et al. [81] shows that BMSCs upregulate DNMT3A and DNMT3A (enzymes responsible for DNA methylation) in MM cells following direct adhesion implying that BMSCs may trigger a global DNA methylation in MM cells; however, this requires further exploration.

### miRNA targeting of p53 signaling pathway: therapeutic applications

Since p53 is in the middle of a highly complex and co-ordinated network involved in challenging any oncogenic input which may jeopardize genome integrity, reactivation of p53 or maintaining its activity seems to be a promising strategy to treat MM. However, this concept bears significance for MM patients carrying wild type but dysfunctional p53, as mtp53 generates a higher propensity for chemoresistance [82]. Consequently, developing powerful strategies to overcome such complication requires exploring the function of mt53 in MM cells. The approaches applied to targeting strategies for the p53 pathway in MM using small molecule compounds (which reportedly activate p53 or restore wild-type activity of mtp53) have been reviewed before [6]. Additionally, limitations concerning such strategies have been put forward as well [83]. Some caveats depending on type and function of the small molecule include DNA damage due to DNA intercalation, independency on p53 of their lethality (or killing effects may not be clearly through p53), inability to trigger post-translational modifications necessary to obtain a p53 response, and finally the sum of all these methodologies does not cover all *TP53* alleles.

Pre-clinical evidences obtained from administration of miRNA mimics (miR-34a [27]) and miR-192, miR-194, 215 [60]), provide promising platforms to support the concept of applying p53-activating miRNAs to MM therapeutic strategies. This strategy will be especially helpful when p53-activating miRNAs are combined with small molecules like nutlin-3a or MI-219 which increase the level of p53 by inhibiting MDM2 [60]. AntagomiRs against miR-125a or miR-125a have not been explored in MM xenograft models yet, but the in vitro observations related to them indicate their potential for translation into in vivo studies. On the other hand, on top of all are still the issues related to application of miRNAs to the clinic [84]. The major concerns are related to delivery strategies to avoid off-target effects, reduced miRNA activity, and production of toxic metabolites. This area demands continuous research to make these small molecules clinically applicable.

### Concluding remarks and future perspectives

In all hematologic malignancies including MM, p53 abnormalities and dysregulation are perhaps the most extensively studied among other critical tumor suppressors or oncogenes. A variety of small molecule compounds with the ability to restore p53 activity have been applied alone or combined with anti-myeloma drugs to MM pre-clinical models yielding promising results [7, 8, 85, 86]. However, MM patients are still dealing with therapy resistance or relapse, which is the most challenging aspect of MM pathogenesis. To that end, contribution of dysfunctional p53 or p53 pathway to MM drug resistance has been insufficiently investigated and demands more detailed research to understand the mechanisms underlying dysregulation of p53 in MM patients associated with disease progression and drug resistance.

Current findings on substantial contribution of miRNAs to tumor suppressor activity of p53 in cancers including MM provide convincing evidence that miRNAs are now part of the p53 regulatory network. That being said, more thorough investigations are still required to establish miRNAs as important regulatory hubs in p53 network to help decipher the mechanism underlying therapy resistance related to dysregulated p53 in MM. Importantly, it is still unknown whether miRNAs have a crucial role in mtp53 function explicitly in MM and whether their effects are dependent on p53 status.

## Conclusion

In conclusion, miRNAs play a key role in p53 network and in the regulation of p53 tumor suppressor activity. Given further comprehensive research, interaction of miRNAs with wtp53 or mtp53 will shed light on the mechanisms underlying drug resistance and relapse in MM. This will indeed provide novel venues for therapeutic targeting of dysregulated p53 pathway in MM and particularly for overcoming drug resistance due to a dysfunctional p53.

## Abbreviations

3′UTR: 3′ untranslated region
ALL: Acute lymphoblastic leukemia
AML: Acute myelogenous leukemia
BCL-2: B cell lymphoma-2
BMF: Bcl-2-modifying factor
BMSCs: Bone marrow stromal cells
BTZ: Bortezomib
CDK4 and CDK6: Cyclin-dependent kinases 4 and 6
CDKN1A: Cyclin-dependent kinase 1 A
CLL: Chronic lymphocytic leukemia
DEX: Dexamethasone
DLBCL: Diffuse large B cell lymphoma
DNMT3A and DNMT3A: DNA (cytosine-5-)-methyltransferase 3 A and B
FISH: Fluorescent in situ hybridization
GADD45: Growth arrest and DNA damage 45
HMCLs: Human myeloma cell lines
KLF13: Kruppel-like factor 13
MDM2: Mouse double minute 2 homolog
MM: Multiple myeloma
mt: Mutant
PCAF: p300-CBP-associated factor
PSMD10: 26S proteasome non-ATPase regulatory subunit 10
PTEN: Phosphatase and tensin homolog
PUMA: p53-upregulated modulator of apoptosis
RISC: RNA-induced silencing complex
SIRT1: NAD-dependent deacetylase sirtuin-1
TSmiR: Tumor suppressor miRNA
wt: Wild type
YY1: Ying Yang 1
